# No genetic causality between obesity and benign paroxysmal vertigo: A two-sample Mendelian randomization study

**DOI:** 10.1515/med-2024-0940

**Published:** 2024-04-05

**Authors:** Zhiyan Guo, Bingyu Huang, Lingxiao Gan, Shanshan Liang, Ying Liu

**Affiliations:** Department of Rehabilitation, The First Affiliated Hospital of Guangxi Medical University, Nanning, Guangxi, China; Department of Rehabilitation, The First Affiliated Hospital of Guangxi Medical University, No. 6 Shuangyong Road, Qingxiu District, Nanning, Guangxi, China

**Keywords:** obesity, benign paroxysmal vertigo, genome-wide association study, Mendelian randomization

## Abstract

**Objective:**

We applied Mendelian randomization to explore the causal relationship between obesity and benign paroxysmal vertigo (BPV).

**Methods:**

We chose two types of obesity diseases. Obesity due to excessive calories and other or unspecified obesity from the FinnGen database. We used genomic significance (*p* < 5 × 10^−8^) to obtain independent single nucleotide polymorphisms (SNPs) as instrumental variables. Similarly, genome-wide association study data for the disease BPV were selected from the FinnGen database. *R* was then used to test the data for multiplicity and heterogeneity, as well as to detect the effect of individual SNPs on the results. Random effects inverse variance weighting was used as the main statistical analysis.

**Results:**

First, by analyzing, we found an outlier in obesity due to excessive calories (rs12956821). Outliers were then removed, and the statistical results were analyzed without heterogeneity (*p* > 0.05) and horizontal pleiotropy (*p* > 0.05), as well as individual SNPs having no effect on the results. Meanwhile, random-effects IVW results showed obesity due to excessive calories (*p* = 0.481; OR = 0.941), and other or unspecified obesity (*p* = 0.640; OR = 0.964).

**Conclusions:**

The present study did not find a causal relationship between the above two obesity types and BPV at the genetic level.

## 1 Introduction

Benign paroxysmal vertigo (BPV) is a common form of vertigo and is also one of the most common forms of peripheral vestibular dysfunction seen in clinical practice. With a lifetime frequency of 2.4%, the illness is categorized as primary or secondary and has a significant recurrence rate [1]. Patients typically experience vertigo and nystagmus symptoms as a result of head position changes [2]. At present, the pathogenesis of BPV is not fully understood, and the etiology of the disease is unknown in most patients and is generally thought to be related to the production of abnormal otoliths in the semicircular canals [3]. Researchers have shown that a number of risk factors, including old age, head trauma, Meniere’s illness, vascular disease, and vitamin D insufficiency, have an impact on the recurrence rate of BPV [4]. Metabolic diseases such as osteoporosis, type I diabetes, hyperlipidemia, gout, and hypertension are strong risk factors for BPV [5]. However, there is currently a lack of consensus and insufficient study on these risk variables [6].

Over the past few decades, there has been a sharp rise in obesity, a metabolic disorder that puts human health at risk [7]. A body mass index of greater than 30 is currently considered the definition of obesity [8]. Excessive caloric consumption, physical activity, and a complex web of social, environmental, and genetic variables all play a role in the development of obesity [9,10]. In people who are genetically predisposed to fat storage, imbalanced energy intake and expenditure often manifest more pronouncedly. This could be due to interactions between brain rewards and homeostatic circuits [11].

Type 1 diabetes, cardiovascular disease, metabolic syndrome, chronic renal disease, hyperlipidemia, hypertension, and several cancers are among the illnesses that obesity increases the risk of developing [12]. Similar to this, a number of metabolic conditions are conducive to the development of BPV [5]. In addition, obesity and BPV seem to be connected in some way based on recent clinical practice observations [13–15]. Determining whether obesity development can increase the likelihood of the occurrence and recurrence of BPV, as well as elucidating the connection between obesity and BPV, becomes crucial. Given the dearth of studies in this area at the moment and the difficulties in running clinical randomized controlled trials. We planned to investigate the genetic basis of the causal relationship between obesity and BPV using Mendelian randomized genome-wide association studies (GWAS) [16–19].

Mendelian randomization (MR) study designs follow Mendel’s laws of inheritance, which state that if a genotype determines a phenotype, then that genotype can be associated with a disease through that phenotype. In order to explore the association between phenotype and disease or between illness and disease, genotype might be employed as an instrumental variable (IV) [20]. Studies using MR can also avoid the effects of confounding variables that were present in earlier clinical studies [21]. GWAS have identified more than 100 independent genes associated with common obesity states in large samples [22]. Genetic variants linked to a higher risk of obesity have been found in several recent GWAS in large patient samples [23]. In order to investigate the relationship between obesity and BPV, two types of obesity (obesity due to excessive calories and other or unspecified obesity) were chosen from the GWAS database.

## 2 Methods

### 2.1 GWAS summary data for obesity due to excess calories and other or unspecified obesity

Maximum GWAS summary data were chosen from the FinnGen database (https://r9.finngen.fi), including obesity due to excessive calories and other or unspecified obesity [24]. The 370,945 participants in the obesity due to excessive calories type of study included 15,045 in the case group, 355,902 in the control group, and a total of 20,170,080 single nucleotide polymorphisms (SNPs). The 366,927 participants in the other type of obesity study were from Europe, with 11,025 in the case group and 355,902 in the control group, for a total of 20,170,026 SNPs. The International Classification of Diseases, Tenth Edition (ICD-10) codes E66.0 and E66.8/E66.9 were used to define two distinct types of obesity, and two distinct types of obesity-associated SNPs were used as genetic IVs, respectively, obesity due to excess calories and other or unspecified obesity. For more information on participants, genotyping, interpolation, and quality control, visit the FinnGen Web site (https://risteys.finregistry.fi/endpoints/E4_OBESITYCAL) and (https://risteys.finregistry.fi/endpoints/E4_OBESITYNAS).

### 2.2 GWAS summary data for BPV

The largest BPV’s GWAS summary data were chosen from the FinnGen database (https://r9.finngen.fi) [24]. The 367,374 European participants in this GWAS included 8,280 in the case group and 359,094 in the control group, for a total of 20,170,074 SNPs. The International Classification of Diseases, 10th edition (ICD-10) codes for H81.8 were used to define all cases. For more information on BPV regarding participants, genotyping, interpolation, and quality control, visit the FinnGen website (https://risteys.finregistry.fi/endpoints/H8_BPV).

### 2.3 Genetic instrumental variants selection

As IVs for the study, two types of obesity (obesity due to excess calories and other or unspecified obesity) were chosen. The IVs chosen for this study satisfy the three hypotheses of MR analysis: IVs and exposure factors are related, IVs are not related to confounders, and IVs influence outcomes through exposure factors. First, we identified important SNPs for both forms of obesity using the genome-wide significance of *p* < 5 × 10^−8^. Then, *r* ^2^ < 0.001 and clumping distance = 10,000 kb were used to eliminate the strong LD-induced chaining imbalance between SNPs. Then, we eliminated SNPs associated with BPV (*p* < 1 × 10^−5^). After conducting a literature search, we discovered the following confounding factors associated with BPV: female gender, hypertension, diabetes, hyperlipidemia, osteoporosis, and vitamin D defenses [4,25,26]. Confounding variables were eliminated using the PhenoScanner database [27]. The intermediate allele frequency palindromic SNPs were then removed. Additionally, we chose SNPs with *F*-statistics >10 as IVs to ensure a greater correlation between IVs and exposure [28]. *F* = *β* ^2^/SE^2^ was used to calculate the *F* statistic [29]. Use the formula *R* ^2^ = (2 × EAF × (1 − EAF) × *β* ^2^)/[(2 × EAF × (1 − EAF) × *β* ^2^) + (2 × EAF × (1 − EAF) × *N* × SE^2^] to calculate *R* ^2^ [30], where *β* represents the estimated genetic influence on obesity and SE represents the genetic influence’s standard error. In the GWAS for the SNP-obesity connection, *N* is the sample size and EAF is the impact allele frequency. In the end, we obtained eight SNPs associated with obesity due to excessive calories and six SNPs associated with other or unspecified obesity (Table 1). Before getting obesity due to excessive calories SNPs, we excluded three palindromic SNPs (rs12507026, rs2076308, rs4517468), six confounding SNPs (rs1446585, rs6567160, rs9928094, rs1861410, rs2867131, rs7189927), and no SNPs associated with BPV existed, and these eight SNPs were used as IV (*F*-statistics >10). Before getting other or unspecified obesity SNPs, we excluded six confounding SNPs (rs11030104, rs7563362, rs10938398, rs538656, rs62048402, rs72892910), the absence of BPV-associated SNPs, and palindromic SNPs, of which six were used as IV (*F*-statistics >10).

**Table 1:** Obesity genetic IVs in benign paroxysmal positional vertigo GWAS

SNP	EA	NEA	EAF	*β*	SE	*p*	*β*	SE	*p*
	**Exposure (obesity due to excess calories) GWAS**						**Outcome (BPV) GWAS**		
rs12956821	G	T	0.359	−0.070	0.012	1.54 × 10^−8^	−0.052	0.017	0.001
rs1595215	G	A	0.230	−0.083	0.014	6.71 × 10^−9^	−0.011	0.019	0.575
rs17587238	A	G	0.080	0.121	0.021	1.05 × 10^−8^	−0.029	0.029	0.324
rs34783010	T	G	0.259	−0.088	0.014	1.23 × 10^−10^	−0.006	0.018	0.748
rs3972483	A	C	0.215	0.096	0.014	1.38 × 10^−11^	−0.014	0.019	0.465
rs5011431	A	G	0.346	0.069	0.012	2.36 × 10^−8^	0.016	0.017	0.327
rs72546309	T	C	0.104	−0.111	0.020	2.90 × 10^−8^	0.026	0.026	0.316
rs826583	G	A	0.214	0.079	0.014	2.88 × 10^−8^	−0.021	0.019	0.285
	**Exposure (Other or unspecified obesity) GWAS**						**Outcome (BPV) GWAS**		
rs10938398	A	G	0.472	0.091	0.014	4.19 × 10^−11^	0.035	0.016	0.029
rs11030104	G	A	0.166	−0.104	0.019	3.59 × 10^−8^	0.032	0.021	0.133
rs13030967	G	A	0.616	0.085	0.014	2.83 × 10^−9^	−0.001	0.016	0.933
rs2300861	T	C	0.545	−0.081	0.014	5.12 × 10^−9^	−0.026	0.016	0.098
rs3734555	A	G	0.331	0.091	0.014	2.76 × 10^−10^	−0.022	0.017	0.185
rs4289073	T	G	0.567	−0.086	0.014	5.14 × 10^−10^	0.016	0.016	0.306
rs67553175	A	G	0.208	0.115	0.017	5.14 × 10^−12^	−0.015	0.020	0.451
rs7563362	G	A	0.854	0.153	0.020	4.42 × 10^−14^	−0.021	0.022	0.351
rs888154	A	C	0.332	−0.086	0.015	6.38 × 10^−9^	−0.001	0.017	0.957

Abbreviations: GWAS, genome‐wide association study; SNP, single‐nucleotide polymorphism; EA, effect allele; NEA, non‐effect allele; EAF, effect allele frequency; *β*, the size of the obesity effect allele’s regression coefficient; SE, standard error; BPV, benign paroxysmal vertigo.

### 2.4 Statistical and MR analysis

#### 2.4.1 Association of obesity genetic IVs with BPV GWAS

From two obesity GWAS pooled datasets, we retrieved eight and nine separate genetic IVs. The results showed a correlation between each genetic IV and the GWAS for BPV (Table 1).

#### 2.4.2 Pleiotropy and heterogeneity test

Using the TwoSampleMR and MRPRESSO packages in R (version 4.3.1), we conducted a two-sample MR analysis of obesity and BPV [31]. MR Egger’s intercept test and the MR-PRESSO method were used to detect horizontal pleiotropy [32,33]. The *p* > 0.05 shows that the IV for the obesity gene does not have horizontal pleiotropy for GWAS for BPV. The Cochran and Rucker *Q* statistic were used to detect heterogeneity in the MR analysis [34,35], with *p* > 0.05 indicating no heterogeneity. The distortion test for MR-PRESSO analysis was used to detect the presence of outliers in our MR analysis, which usually have an impact on the heterogeneity of the data. We need to reanalyze the results after removing the outliers [32].

#### 2.4.3 MR analysis

The mr_egger, weighted median, inverse variance weighting (IVW), simple mode, and weighted mode methods were used to analyze the causal relationship between obesity and BPV. We mainly used the results of IVW as the primary basis [36]. The mr_egger, weighted median, simple mode, and weighted mode methods were used as a basis for ancillary judgments [37,38]. Obesity and BPV are causally related when *p* < 0.05.

#### 2.4.4 Single SNP effect analysis

We used “mr_scatter_plot” in R to verify the causal relationship between obesity and BPV [39]. “mr_forest_plot” was used to determine individual SNP effect sizes in the effect of obesity on BPV [40]. The “mr_leaveoneout_plot” sensitivity analysis to determine whether the relationship between obesity and BPV is affected by each SNP [41].


**Ethics approval and consent to participate:** Ethical review approval has been obtained for the Finnish study. The present study was analyzed using information from its database. No additional ethical review approval was required.

## 3 Results

### 3.1 Pleiotropy and heterogeneity of obesity genetic IVs

First, in the MR analysis between obesity due to excess calories and BPV by MR-PRESSO, we found an outlier (rs12956821). The analysis between other obesity or obesity of unknown type and BPV did not show any outliers. Finally, the analysis of the results of removing outliers continues. The results showed no horizontal pleiotropy or heterogeneity for each obesity type and BPV (Table 2).

**Table 2:** Tests of pleiotropy and heterogeneity of genetic instrumental variants for two types of obesity

mr_egger			PRESSO	mr_egger			IVW		
Intercept	SE	*p*	*p*	*Q*	*Q*_df	*p*	*Q*	*Q*_df	*p*
**Pleiotropy test (obesity due to excess calories genetic IVs)**				**Heterogeneity test (obesity due to excess calories genetic IVs)**					
0.068	0.045	0.192	0.628	2.268	5	0.811	18.871	6	0.605
**Pleiotropy test (other or unspecified obesity genetic IVs)**				**Heterogeneity test (other or unspecified obesity genetic IVs)**					
0.071	0.063	0.321	0.362	4.278	4	0.370	5.650	5	0.342

Abbreviations: GWAS, genome‐wide association study; IVW, inverse variance weighted; MR, Mendelian randomization; SE, standard error.

*p* > 0.05 represents no significant pleiotropy and heterogeneity.

### 3.2 Obesity was not associated with BPV

We used MR analysis to determine the causal relationship between the two types of obesity and BPV at the gene level after removing the outliers. IVW (*β* = −0.060; *p* = 0.481; odds ratio [OR] = 0.941; 95% confidence interval [95% CI]: 0.796–1.113) analyzed the causality results of obesity due to excess calories and BPV, and other tests similarly supported IVW’s results. IVW (*β* = −0.037; *p* = 0.640; OR = 0.964; 95% CI: 0.825–1.125) analyzed the causality results of other or unspecified obesity and BPV, and other tests similarly support IVW’s results (Table 3). In summary, our analyses show that there is no connection between genetic alterations in obesity brought on by obesity due to excess calories and other or unspecified obesity for BPV.

**Table 3:** Causal association of obesity with BPV

Method	*N*	*β*	SE	*p*	OR	95% CI
**Obesity due to excess calories**						
mr_egger	7	−0.821	0.512	0.170	0.440	0.161–1.201
Weighted median	7	−0.106	0.108	0.326	0.899	0.728–1.111
IVW	7	−0.060	0.085	0.481	0.941	0.796–1.113
Simple mode	7	−0.224	0.179	0.258	0.800	0.563–1.136
Weighted mode	7	−0.213	0.176	0.273	0.808	0.572–1.142
**Other or unspecified obesity**						
mr_egger	6	−0.843	0.703	0.296	0.430	0.109–1.705
Weighted median	6	−0.091	0.093	0.327	0.913	0.760–1.096
IVW	6	−0.052	0.081	0.521	0.949	0.809–1.113
Simple mode	6	−0.133	0.147	0.408	0.876	0.656–1.169
Weighted mode	6	−0.137	0.139	0.368	0.872	0.664–1.144

Abbreviations: IVW, inverse variance weighted; MR, Mendelian randomization; *N*, number of single‐nucleotide polymorphism; *β*, the size of the obesity effect allele’s regression coefficient; SE, standard error; OR, odds ratio; 95%CI, 95% confidence interval; BPV, benign paroxysmal vertigo. *p* < 0.05 indicates a causal relationship between obesity and BPV.

### 3.3 No significant bias in single obesity SNP effect

**Figure 1:**
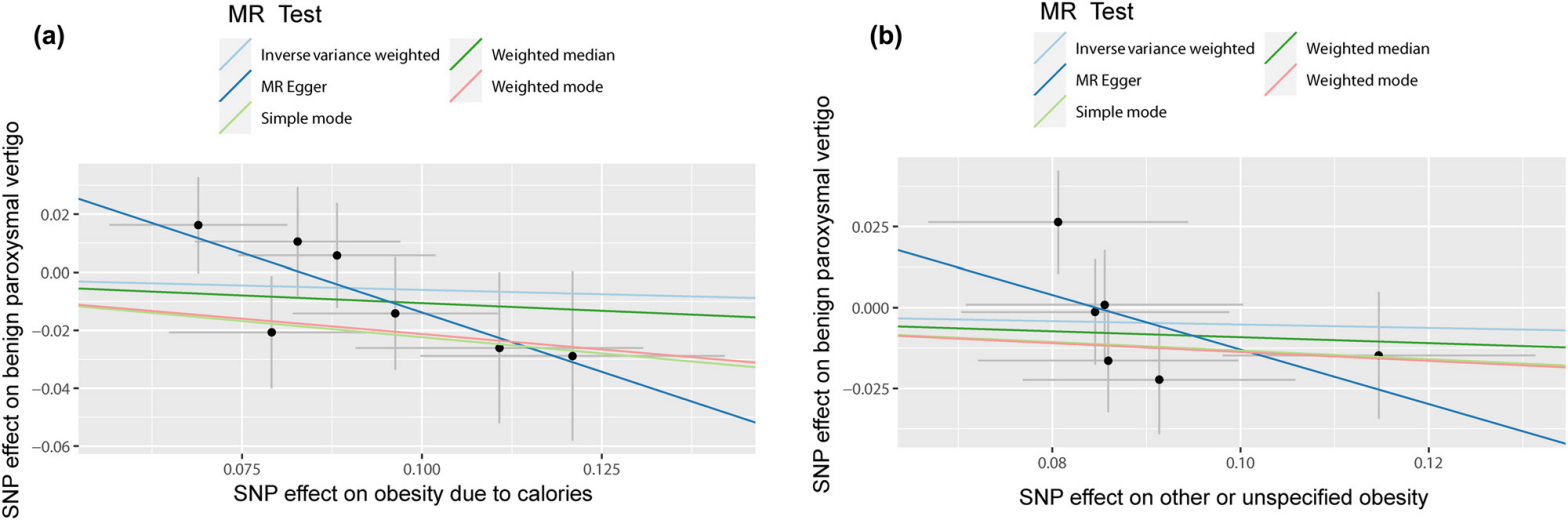
Individual estimates about the putative causal effect of obesity on BPV. (a) Obesity due to excess calories and benign paroxysmal vertigo. (b) Other or unspecified obesity and benign paroxysmal vertigo. IVW, inverse variance weighted; MR, Mendelian randomization; SNP, single‐nucleotide polymorphism. BPV, benign paroxysmal vertigo.

**Figure 2:**
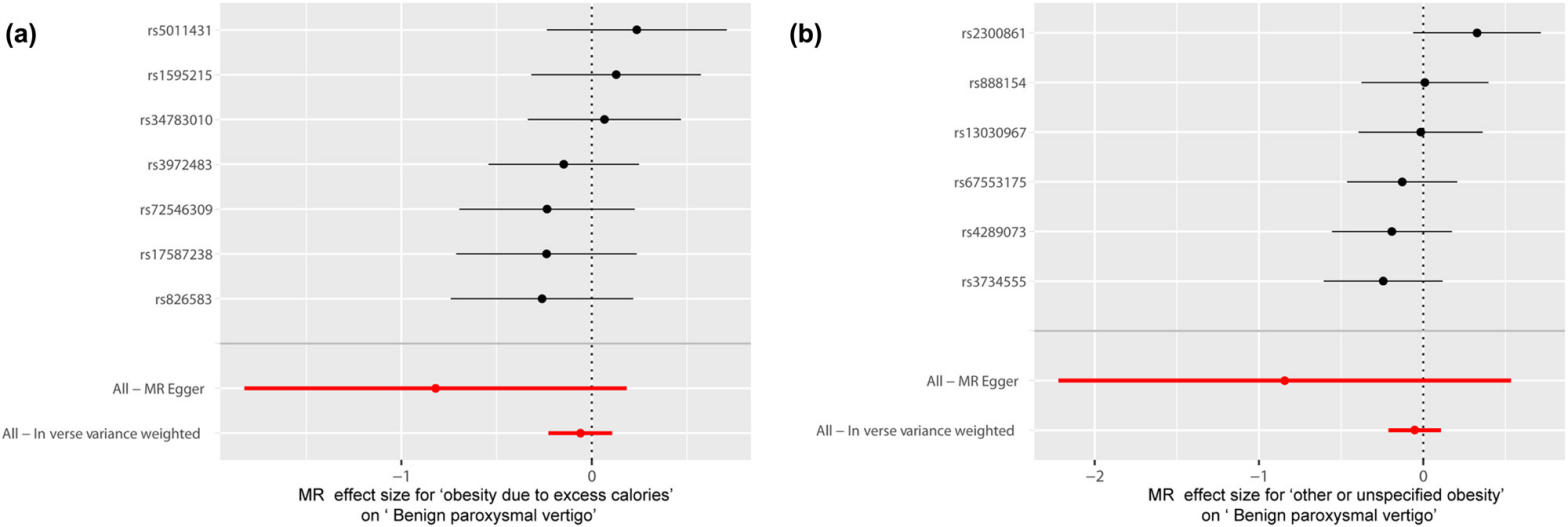
Forest plot of obesity associated with BPV. (a) Obesity due to excess calories and benign paroxysmal vertigo. (b) Other or unspecified obesity and benign paroxysmal vertigo. MR, Mendelian randomization. BPV, benign paroxysmal vertigo.

**Figure 3:**
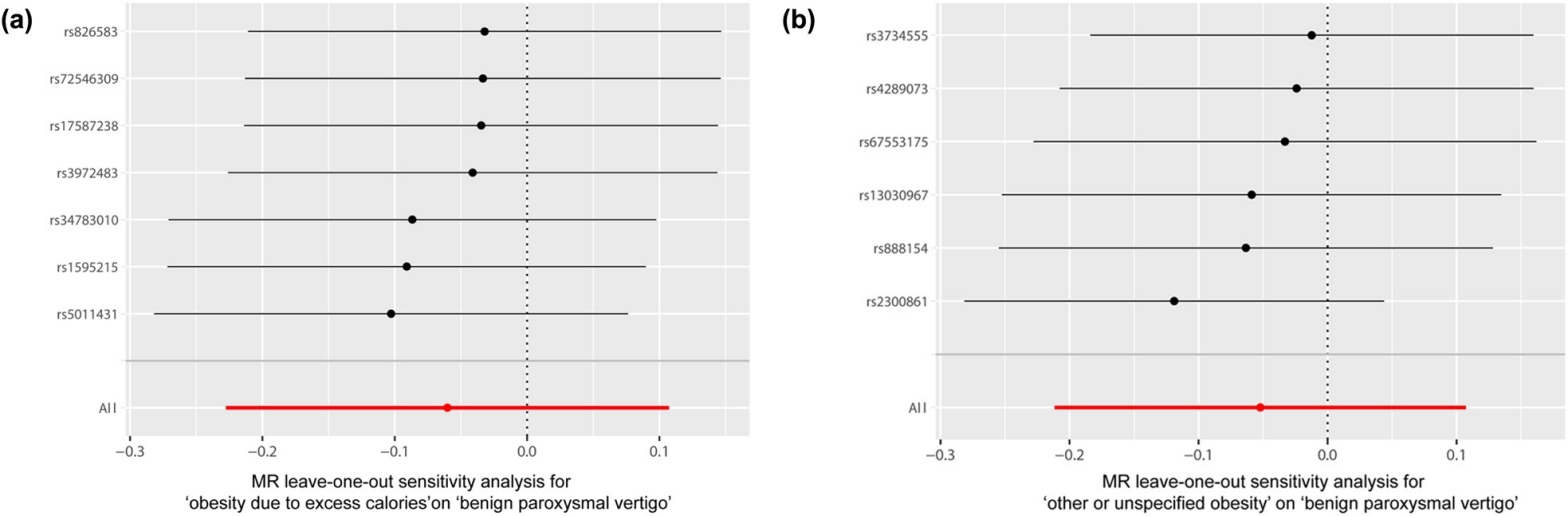
MR leave‐one‐out sensitivity analysis for the effect of obesity SNPs on BPV. (a) Obesity due to excess calories and benign paroxysmal vertigo. (b) Other or unspecified obesity and benign paroxysmal vertigo. MR, Mendelian randomization; SNP, single‐nucleotide polymorphism. BPV, benign paroxysmal vertigo.

It was shown by MR analysis that the effect of each SNP change on obesity does not have an effect on BPV (Figure 1). The same is demonstrated for the studies of the individual SNP effect values (Figure 2). At the same time, the leave-one-out sensitivity analysis we performed showed that the final results did not receive an effect when we removed any obese SNP (Figure 3).

## 4 Discussion

In this study, we explored the causal relationship between obesity and BPV at the genetically inherited level. After effective screening of SNPs and removal of outliers using MR and using obesity-associated SNPs as our genetic IVs, we analyzed that there was no horizontal pleiotropy (*p* > 0.05) or heterogeneity (*p* > 0.05) in the SNPs for the two obesity types and BPV, respectively. Meanwhile, we found that all five MR analyses, mainly IVW, showed that the two types of obesity, obesity due to excess calories and other or unspecified obesity, respectively, were not causally correlated genetically with BPV (*p* > 0.05).

Clinically, we observe that patients with BPV tend to be elderly, female, and have metabolic disorders such as diabetes mellitus, hypertension, hyperlipidemia, and osteoporosis [42]. These factors that contribute to BPV are commonly associated with the onset and progression of obesity. Despite the fact that there is little in-depth literature on the subject, there is also no literature that negates the existence of a relationship between the two. Only a few observational studies have explored the existence of a relationship and the fact that obesity is sometimes used as a confounding factor in exploring vertigo with other disorders [43–46]. At the same time, it is widely recognized in research that obesity is a high-risk predisposing factor for a variety of diseases. Therefore, there has been ambiguity in defining whether there is some association between obesity and BPV and the magnitude of the association. Limitations in conducting clinical trials exploring the relationship seem to have created an obstacle in resolving this issue. In recent years, with the widespread use of MR experiments, we have found ideas to solve the problem. Since parental alleles are randomly assigned to offspring, single nucleotide site variants can be more appropriately used to genetically explore the relationship between obesity and BPV [47]. However, at the genetic level, this study did not find a significant causal relationship between the two. BPV is one of the many vertigo disorders; other vertigo disorders include vestibular neuritis, Meniere’s disease, central vertigo, and others [48]. Similarly, obesity, as we know it, possesses multiple types. It has been shown that obesity negatively affects residual dizziness in BPV patients after otolith repositioning operations [13]. Additionally, in studies of adults with and without dizziness or vertigo, the difference in body mass index and obesity was found to be significantly higher in the group with dizziness [14]. Thus, some studies have observed an association between obesity and vertigo. Metabolic disorders such as hypertension, diabetes, and hyperlipidemia associated with obesity can cause cerebrovascular atherosclerosis as well as the development of insufficient arterial blood supply, which can cause vertigo. There are no more refined studies exploring the relationship between BPV and obesity at the clinical and genetic levels. In this study, BPV, obesity due to excess calories, and other or unspecified obesity were selected as relatively common types of vertigo and obesity. The relationship between BPV and obesity due to excess calories and other or unspecified obesity was explored. The number of obese patients is increasing today, and the adverse effects on them are manifold. Although this study did not conclude that there is a genetic and hereditary association between obesity and BPV, we cannot ignore other clinical effects that obesity may have on the occurrence and recurrence of BPV.

## 5 Limitations

There are also some limitations to this study. First, since the gene IVs for obesity and the GWAS for BPV are of European origin, the results of the study may not necessarily be genetically relevant for populations of other origins. Second, since only the two most common types of obesity (obesity due to excess calories and other or unspecified obesity) were selected in this study, it does not have much significance as a guide for other types of obesity, such as drug-induced obesity and obesity hypoventilation syndrome. Next, future clinical trials are necessary in this study to further elucidate that there is no causal relationship between obesity and BPV.

## 6 Conclusion

Although our analyses did not indicate a causal relationship between obesity due to excess calories and other or unspecified obesity and BPV in terms of genetic inheritance, we cannot rule out the existence of some unknown relationship between the two at the non-genetic level. Therefore, a broader and deeper investigation of the relationship is needed in the future.

## Abbreviations

BPV: benign paroxysmal vertigo
EAF: effect allele frequency
GWAS: genome-wide association study
IVs: instrumental variables
IVW: inverse variance weighting
MR: Mendelian randomization
OR: odds ratio
SE: standard error
SNPs: single nucleotide polymorphisms
95% CI: 95% confidence interval
